# Optimizing Hydrogen Binding on Ru Sites with RuCo Alloy Nanosheets for Efficient Alkaline Hydrogen Evolution

**DOI:** 10.1002/anie.202113664

**Published:** 2021-12-07

**Authors:** Chao Cai, Kang Liu, Yuanmin Zhu, Pengcheng Li, Qiyou Wang, Bao Liu, Shanyong Chen, Huangjingwei Li, Li Zhu, Hongmei Li, Junwei Fu, Yu Chen, Evangelina Pensa, Junhua Hu, Ying‐Rui Lu, Ting‐Shan Chan, Emiliano Cortés, Min Liu

**Affiliations:** ^1^ School of Physics and Electronics Central South University Changsha 410083 P. R. China; ^2^ Department of Materials Science and Engineering Southern University of Science and Technology Shenzhen 518055 P. R. China; ^3^ Nanoinstitut München Fakultät für Physik Ludwig-Maximilians-Universität München 80539 München Germany; ^4^ School of Materials Science and Engineering Zhengzhou University Zhengzhou 450001 P. R. China; ^5^ National Synchrotron Radiation Research Center Hsinchu 300 Taiwan

**Keywords:** Alkaline HER, Cobalt nanosheet, Hydrogen adsorption/desorption, Orbital modulation, Ruthenium

## Abstract

Ruthenium (Ru)‐based catalysts, with considerable performance and desirable cost, are becoming highly interesting candidates to replace platinum (Pt) in the alkaline hydrogen evolution reaction (HER). The hydrogen binding at Ru sites (Ru−H) is an important factor limiting the HER activity. Herein, density functional theory (DFT) simulations show that the essence of Ru−H binding energy is the strong interaction between the $4d_{z^{2}}$ orbital of Ru and the 1s orbital of H. The charge transfer between Ru sites and substrates (Co and Ni) causes the appropriate downward shift of the $4d_{z^{2}}$ ‐band center of Ru, which results in a Gibbs free energy of 0.022 eV for H* in the RuCo system, much lower than the 0.133 eV in the pure Ru system. This theoretical prediction has been experimentally confirmed using RuCo alloy‐nanosheets (RuCo ANSs). They were prepared via a fast co‐precipitation method followed with a mild electrochemical reduction. Structure characterizations reveal that the Ru atoms are embedded into the Co substrate as isolated active sites with a planar symmetric and Z‐direction asymmetric coordination structure, obtaining an optimal $4d_{z^{2}}$ modulated electronic structure. Hydrogen sensor and temperature program desorption (TPD) tests demonstrate the enhanced Ru−H interactions in RuCo ANSs compared to those in pure Ru nanoparticles. As a result, the RuCo ANSs reach an ultra‐low overpotential of 10 mV at 10 mA cm^−2^ and a Tafel slope of 20.6 mV dec^−1^ in 1 M KOH, outperforming that of the commercial Pt/C. This holistic work provides a new insight to promote alkaline HER by optimizing the metal‐H binding energy of active sites.

## Graphical abstract

Optimizing Ru−H adsorption/desorption efficiency, via adjusting the Ru $4d_{z^{2}}$ orbital in RuCo alloy‐nanosheets, enables highly promoted alkaline hydrogen evolution reaction. This optimized adsorption/desorption efficiency is demonstrated by the hydrogen sensor and temperature programmed desorption experiments. The RuCo alloy‐nanosheets possess a record low overpotential of 10 mV at 10 mA cm^−2^, superior to the commercial Pt/C and Ru/C.

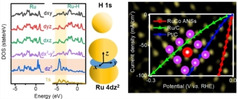

The alkaline electrocatalytic hydrogen evolution reaction (HER) is an attractive strategy to realize efficient conversion of electric energy to chemical energy.[^1^, ^2^, ^3^, ^4^, ^5^, ^6^] Pt is recognized as the most efficient catalyst for HER. However, the scarcity and high‐cost of Pt greatly impedes its practical applications.[^7^, ^8^, ^9^] Ru is a promising substitute for Pt due to its low‐cost (one twenty‐fifth of Pt) and similar Gibbs free energy change of H* (Δ*G*_H*_) to that of Pt.[^10^, ^11^, ^12^, ^13^, ^14^, ^15^, ^16^] Unfortunately, the sluggish hydrogen chemical adsorption/desorption on Ru (H*+H*→H_2_+2*), caused by the inappropriate Ru−H binding energy, vastly limit its HER performance.[^5^, ^17^] Generally, too weak Ru−H binding energy results in a poor proton adsorption during HER, while too strong Ru−H binding energy will poison the Ru sites and thus hinder the H_2_ formation.^[18]^ Though numerous efforts have been made to promote the HER activity of Ru‐based catalysts, such as alloy,[^7^, ^16^, ^19^] sulfide,[^20^, ^21^, ^22^, ^23^, ^24^] and phosphides,[^25^, ^26^, ^27^, ^28^, ^29^] etc., the intrinsic relationship between Ru−H binding energy and HER activity is still ambiguous. Revealing the essential interaction between Ru and H in Ru−H bonds and the accurate regulation of Ru−H binding energy have become a potential strategy to achieve better HER performance.

The Ru−H binding energy is determined by the strong interaction between the Ru 4d orbital and the H 1s orbital. Adjusting the Ru 4d electronic orbital structure is a direct way to optimize the hydrogen binding energy on Ru sites.[^25^, ^30^, ^31^] The interaction and charge transfer between the ambient atoms and the Ru sites can obviously affect the 4d electronic orbital structure of Ru.[^10^, ^17^, ^19^, ^32^] Nonmetallic atoms, such as S and P,[^33^, ^34^, ^35^, ^36^, ^37^] can extract electrons from Ru sites,[^20^, ^25^, ^38^] and then change the 4d electronic orbital structure of Ru and Ru−H binding energy. Due to the over‐modulation—caused by the large amount of charge transfer between non‐metal atoms and Ru—it is difficult to achieve accurate tuning of Ru−H binding energy. Mild interaction usually happens between different metals. Designing Ru−M active sites (M represents metals) can be a reliable way for achieving a precise Ru−H modulation. For example, Zhang et al. reported the Mo‐modified Ru sites with enhanced HER activity.^[41]^ Chen et al. reported that the Ru−H can be regulated using Au as electron donor of Ru sites.^[42]^ The cheap transition metals Co and Ni are widely used catalysts for catalysis applications,[^43^, ^44^, ^45^, ^46^, ^47^, ^48^, ^49^, ^50^, ^51^, ^52^] and their electronegativities are 1.88 and 1.91 respectively. Compared with the electronegativity of 2.20 for Ru, they are potential candidates for regulating the electronic structure of Ru site. Reasonable construction of Ru−Co(Ni) catalysts with clear site structure can optimize HER activity by regulating the hydrogen binding on Ru sites.

In this work, density functional theory (DFT) studies reveal that the interaction between the Ru $4d_{z^{2}}$ orbital and H 1s orbital determines the Ru−H binding energy. It is the most direct way to optimize the Ru−H binding energy by influencing the vertical Z direction Ru $4d_{z^{2}}$ orbital. In the alloy system, Ru atoms are embedded in the metal matrix and coordinated symmetrically in the plane direction. Due to the asymmetry in the Z direction, the charge transfer between the metal matrix and Ru sites can directly affect the charge arrangement of the $4d_{z^{2}}$ orbital of Ru, thus affecting the Ru−H binding energy and HER activity. DFT calculations systematically compared the charge transfer between Ru and Co or Ni matrix, the $4d_{z^{2}}$ ‐band center of Ru, and the Ru−H binding energy. The Δ*G*_H*_ at the Ru site on the RuCo alloy is optimized from 0.133 to 0.022 eV. Based on these theoretical calculation results, we synthesized ultra‐thin Co nanosheets, and then embedded Ru sites in Co nanosheets to construct RuCo alloy‐nanosheets (RuCo ANSs). Structure characterization confirmed the structure model of Ru sites in Co nanosheets. The planar symmetric and Z‐direction asymmetric coordination structure of Ru sites in the Co nanosheets makes it possible to accurately optimize the Ru $4d_{z^{2}}$ direction electronic structure. Hydrogen sensor and temperature program desorption (TPD) tests demonstrated the enhanced Ru−H interactions in RuCo ANSs compared to those in pure Ru nanoparticles. As expected, the prepared RuCo ANSs exhibit a superior HER performance in 1.0 M KOH with a low overpotential of 10 mV at 10 mA cm^−2^, and a low Tafel slope of 20.6 mV dec^−1^. Its turnover frequency (TOF) is twice that of commercial Pt/C and 15‐fold of commercial Ru/C at an overpotential of 10 mV. The long‐term HER measurement and surface structure characterization verify that the RuCo ANSs possess high stability in HER.

Firstly, we constructed a Ru structure model to investigate the essential interaction between Ru and H in Ru−H bonds. Hydrogen (H*) possesses two adsorption configurations on the surface of Ru, namely the top site and the hollow site (Figure S1a). The Gibbs free energy change of H* (Δ*G*_H*_) at the top and hollow adsorption sites is 0.133 and −0.245 eV, respectively (Figure S1b), indicating that the weak adsorption of H* on the Ru top site restricts HER. We further studied the electronic orbital structure of Ru 4d and H 1s at the top adsorption configuration. As shown in Figure 1a, the Ru $4d_{z^{2}}$ orbital clearly changes after hydrogen adsorption. These results indicate that the Ru $4d_{z^{2}}$ orbital contributes largely to the Ru−H interaction. The illustration in Figure 1a shows a schematic of the coupling of the Ru $4d_{z^{2}}$ with H 1s orbitals. Adjusting the Z direction electronic structure of the Ru $4d_{z^{2}}$ orbital is the most direct strategy to optimize the Ru−H binding energy.

**Figure 1:**
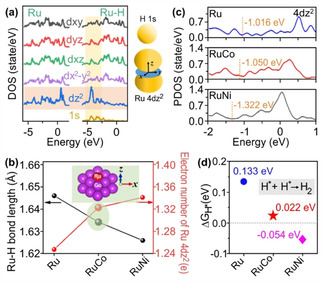
Theoretical calculation results. a) The PDOS of Ru 4d orbitals before and after H* adsorption. The inset shows the schematic diagram of orbital coupling between Ru $4d_{z^{2}}$ and H 1s. b) The Ru−H bond lengths and electron numbers of Ru $4d_{z^{2}}$ in Ru, RuCo and RuNi models. The inset shows the RuCo model planar symmetric and Z‐direction asymmetric coordination structure. c) The band center positions of Ru $4d_{z^{2}}$ in Ru, RuCo and RuNi models. d) The H* Gibbs free energy for Ru, RuCo and RuNi models. The dotted line corresponds to a Gibbs free energy equal to zero.

In order to analyze the adjusted electronic structure of Ru $4d_{z^{2}}$ , the structure models of Ru embedded in Co and Ni with symmetry in the xy plane and asymmetry in the Z direction were constructed (Figure S2a). The ΔG_H*_ at different adsorption sites and crystal planes were compared. The RuCo(100) and RuNi(100) with top adsorption sites exhibit the better HER performance (Figures S2b–f). To explore the correlation between HER performance and Ru−H binding energy, we analyzed the Ru−H bond lengths, charge transfer in Ru, and Δ*G*_H*_ variations of hydrogen formation on Ru sties. Figure 1b collects the Ru−H bond lengths of Ru, RuCo, and RuNi, which are sorted in order. The shorter bond length indicates the stronger Ru−H interaction. The bond length variations are usually correlated to the charge redistribution, hence, we analyzed the charge state of Ru on various supports. Figure S3 compares the charge transfer between the Ru site and substrates in different models. The numbers of total transferred electrons are −0.057, 0.121 and 0.051 e in Ru, RuCo, and RuNi, respectively. Clearly, there is no correlation between the number of total transferred electrons and the Ru−H interaction. Figure S4 shows the projected density of states (PDOS) analysis of Ru sites. The number of Ru $4d_{z^{2}}$ occupied electrons are 1.25, 1.32, and 1.34 e in Ru, RuCo, and RuNi, respectively (Figure 1b). The Ru $4d_{z^{2}}$ band centers of Ru, RuCo, and RuNi are calculated to be −1.016, −1.050, and −1.322 eV (Figure 1c), respectively, directly related to the hydrogen adsorption on the Ru sites. These results indicate that the electronic environment of Ru $4d_{z^{2}}$ makes the direct contribution to the Ru−H interaction. RuCo and RuNi alloy structures can influence the Ru−H interaction by regulating the Ru $4d_{z^{2}}$ electron environment, resulting in better HER performance. As shown in Figure 1d, with the fine adjustment of the Ru $4d_{z^{2}}$ orbital, the Δ*G*_H*_ on the Ru site of RuCo can be changed from 0.133 eV to 0.022 eV, where Δ*G*_H*_ of RuNi is −0.054 eV. These results demonstrate the superiority of adjusting the Ru $4d_{z^{2}}$ orbital using Co as support to achieve optimized Ru−H.

Inspired by the theoretical calculation results, we synthesized RuCo ANSs. The detailed formation process of RuCo ANSs is illustrated in Figure S5. Firstly, Ru incorporated ultrathin Co nanosheets (as‐prepared RuCo precursor) were prepared by the fast co‐precipitation method. Then, the RuCo ANSs were obtained by a mild in‐situ electrochemical reduction process. A series of RuCo alloy‐nanosheets with different proportions were prepared by adjusting the Ru−Co feeding ratio, and their HER properties were tested. As shown in Figure S6, HER performances are related to the ratio of Ru to Co. The actual ratio of Ru to Co in the samples was determined by inductively coupled plasma optical emission spectrometry (ICP‐OES) (Table S1), and the optimal ratio was found to be 1 : 3.06.

Figures S7 and S8 compare the X‐ray diffraction (XRD) patterns and X‐ray photoelectron spectroscopy (XPS) spectra of the as‐prepared RuCo precursor and RuCo ANSs. The XRD pattern of RuCo ANSs exhibits metal Co signals (PDF#15‐0806), Ru signals (PDF#06‐0663), and RuCo alloy signals (PDF#65‐8976), verifying the RuCo alloy formation. Correspondingly, the binding energies of Co and Ru are clearly negatively shifted to the metal state after electrochemical reduction in the XPS spectra. Figure S9 exhibits the Raman spectra of these samples. A clear signal for Co_3_O_4_ oxides can be observed in the as‐prepared RuCo precursor, but not in the RuCo ANSs sample. These results indicate the formation of RuCo alloy in RuCo ANSs.

The scanning electron microscopy (SEM) image demonstrates the two‐dimension nanosheet morphology of RuCo ANSs (Figure 2a). The scanning transmission electron microscopy (STEM) image (Figure 2b) shows the cross‐section of the ultrathin layered structure in RuCo ANSs. Both the STEM‐energy dispersed spectroscopy (STEM‐EDS) mapping of the RuCo precursor (Figure S10) and RuCo ANSs (Figure S11) show that the Ru in samples homogeneously distributes in the Co matrix. The small Ru cluster can be observed. The atomic force microscopy (AFM) results show that the thickness of the RuCo ANSs is ca. 1.8 nm, as expected for the layered structure (Figures S11a,b). The fast Fourier transform (FFT) image shows the Ru, RuCo and Co signal (Figure S11c and 11d). The Ru sites trapped into the Co matrix in RuCo ANSs are confirmed by a high‐resolution STEM image (Figure 2c).To clarify the arrangement of the Ru atoms in the Co matrix, a linear analysis was conducted. As shown in Figure 2d, the profiles of area 1 verify the Ru substitution in the Co lattice. The lattice distance of 0.18 nm (marked by the black parallel lines) is assigned to fcc Co (200), consistent with the XRD pattern in Figure S7.

**Figure 2:**
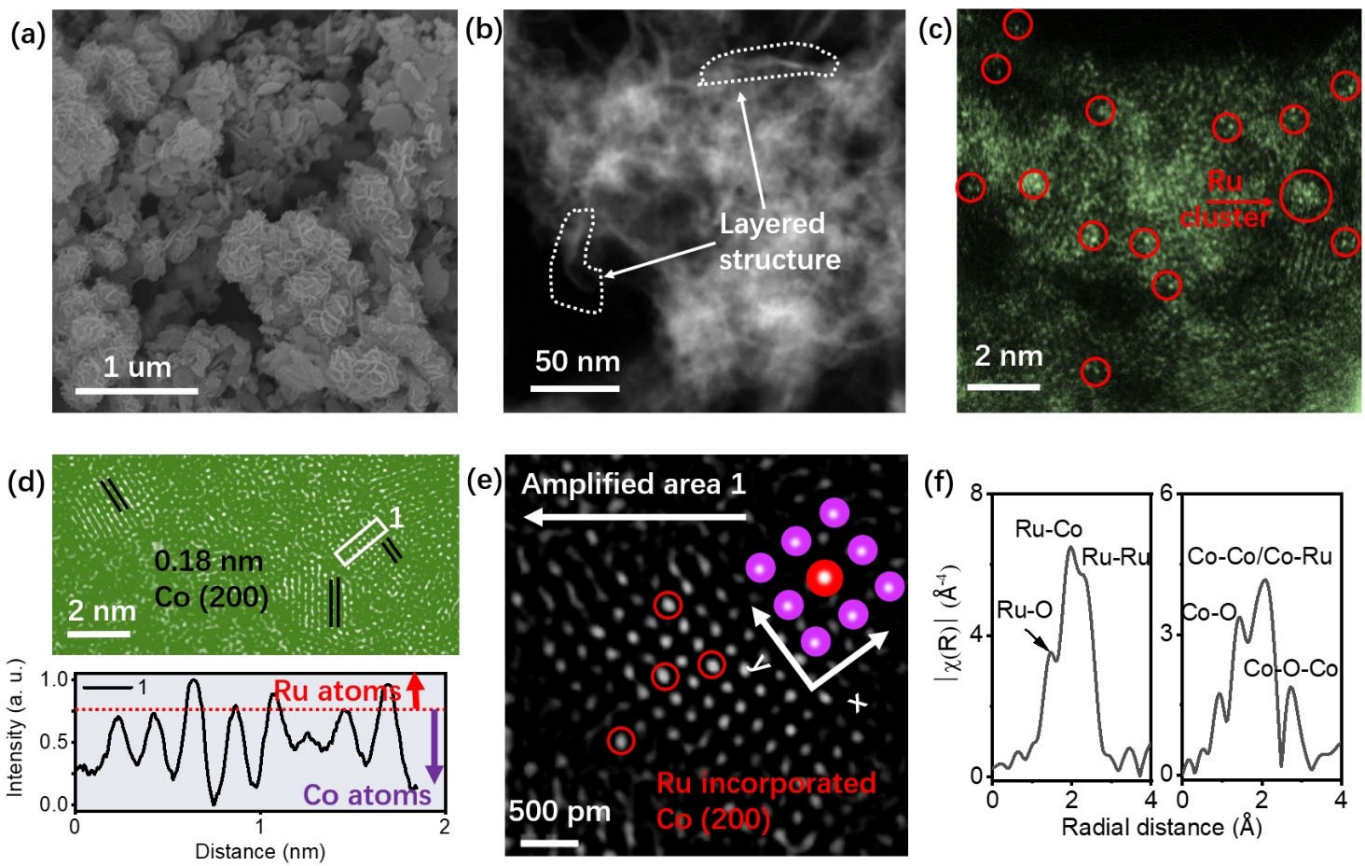
Structural characterization of RuCo ANSs. a) SEM image showing a two‐dimensional nanosheet morphology. b) STEM image. c) High‐resolution STEM image. d) Atomic resolution STEM image (top) and relative line profile (bottom). e) Atomic structure of Ru‐substituted Co (200). The image is an amplification at the white rectangle 1 in image d. The red circles in e mark the Ru sites. f) Ru K‐edge (left) and Co K‐edge (right) EXAFS.

To get further insight into the atomic structure of RuCo ANSs, the atomic resolution high angle annular dark‐field‐STEM (HAADF‐STEM) characterization was performed. As shown in Figure 2e, the Ru and Co atomic model in RuCo ANSs is successfully determined according to the fine structure analysis, where the Ru atoms are atomically incorporated into the Co (200) facet with the xy planar symmetric structure. The extended X‐ray absorption fine structure (EXAFS) spectra of RuCo ANSs (Figures 2f, and S12, S13) show the existence of strong Ru−Co and Co−Ru bonding peaks, further verifying the Ru incorporation into the Co structure. The Ru K‐edge of RuCo ANSs shows three peaks at 2.02 Å, 2.54 Å, and 2.64 Å (Figure 2f and Figure S13c), corresponding to Ru−O, Ru−Co, and Ru−Ru, respectively. This demonstrates the RuCo alloy formation. Similar results also can be observed in the Co K‐edge in Figure 2f and Figure S13d. These results are consistent with the STEM and XRD results. The Ru−O and Co−O peaks could have resulted from air exposure. Table S2 shows the relevant parameters in the EXAFS fitting process. The above multitechnique structural characterization proves that the RuCo ANSs structure is consistent with the theoretical model, that is, the Ru sites have a xy plane symmetric structure and an asymmetric coordination structure in the Z direction.

Next, the catalytic activities of RuCo ANSs were recorded. Figure 3a compares the polarization curves of the optimal RuCo ANSs and related Ru‐based and Pt‐based catalysts. Clearly, the RuCo ANSs show the best HER activity, even better than commercial 20 wt % Ru/C and Pt/C. The corresponding Tafel slopes and overpotentials (@10 mA cm^−2^) were calculated and are shown in Figure 3b and c. The RuCo ANSs exhibit a Tafel slope of 20.6 mV dec^−1^ (Figure 3b) and an overpotential of 10 mV at 10 mA cm^−2^ (Figure 3c). The Tafel value is lower than 33 mV dec^−1^, verifying that the H* adsorption/desorption is the rate‐determining step of RuCo ANSs during HER. The ultra‐low overpotential of 10 mV is competitive with the catalysts reported to date (Table S3). Figure 3d shows that the turnover frequency (TOF) value of RuCo ANSs is 8.528 s^−1^ at 100 mV, which is twice that of commercial 20 wt % Pt/C (3.527 s^−1^), 15‐fold of Ru/C (0.555 s^−1^), and 1065‐fold of Co (0.008 s^−1^). Moreover, the electrochemical impedance spectra (EIS) show that RuCo ANSs possess the highest charge transport efficiency and comparable to Ru/C and the RuCo precursor (Figure S14), verifying the excellent H* adsorption/desorption efficiency on RuCo ANSs for better interface charge transfer.

**Figure 3:**
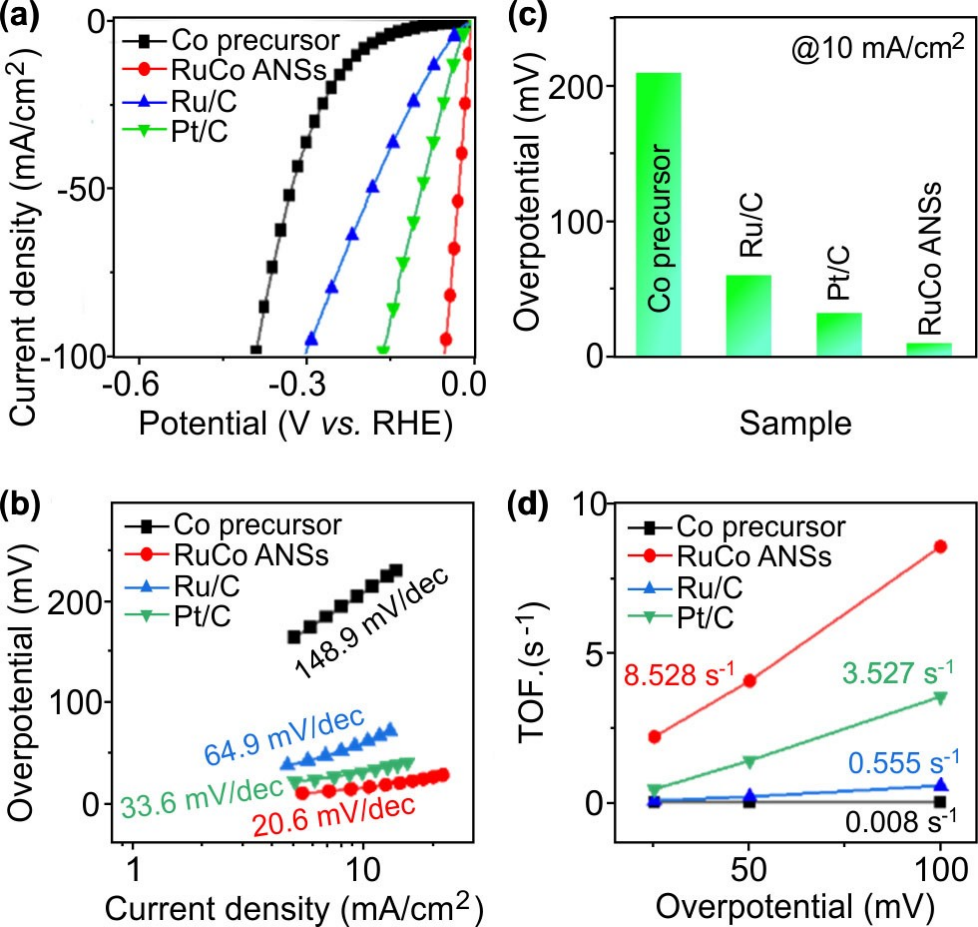
Electrochemical HER performance. Polarization curves (a), Tafel plots (b), calculated overpotentials at 10 mA cm^−2^ (c), and TOF values (d) of various catalysts.

Stability is a very important factor for estimating the catalysts cost in practical applications. To test the stability of RuCo ANSs, their chronoamperometry response was examined (Figure 4a). The RuCo ANSs show high stabilities at 10 mA cm^−2^ (−10 mV), 180 mA cm^−2^ (−100 mV), and 320 mA cm^−2^ (−140 mV), respectively. More importantly, the RuCo ANSs still show high stability after these long‐term catalytic tests, proving the high catalytic activity stability of RuCo ANSs during harsh HER process. Cyclic voltammetry was also used for catalytic stability testing (Figure 4b), where the RuCo ANSs possess an overpotential decay of 2 mV (10 mA cm^−2^) after 10k cycles. After tests, an ignorable Ru signal was detected in the electrolyte by ICP‐OES (Table S1), demonstrating the high stability of RuCo ANSs. These results further prove that the RuCo ANSs can work well under complex conditions.

**Figure 4:**
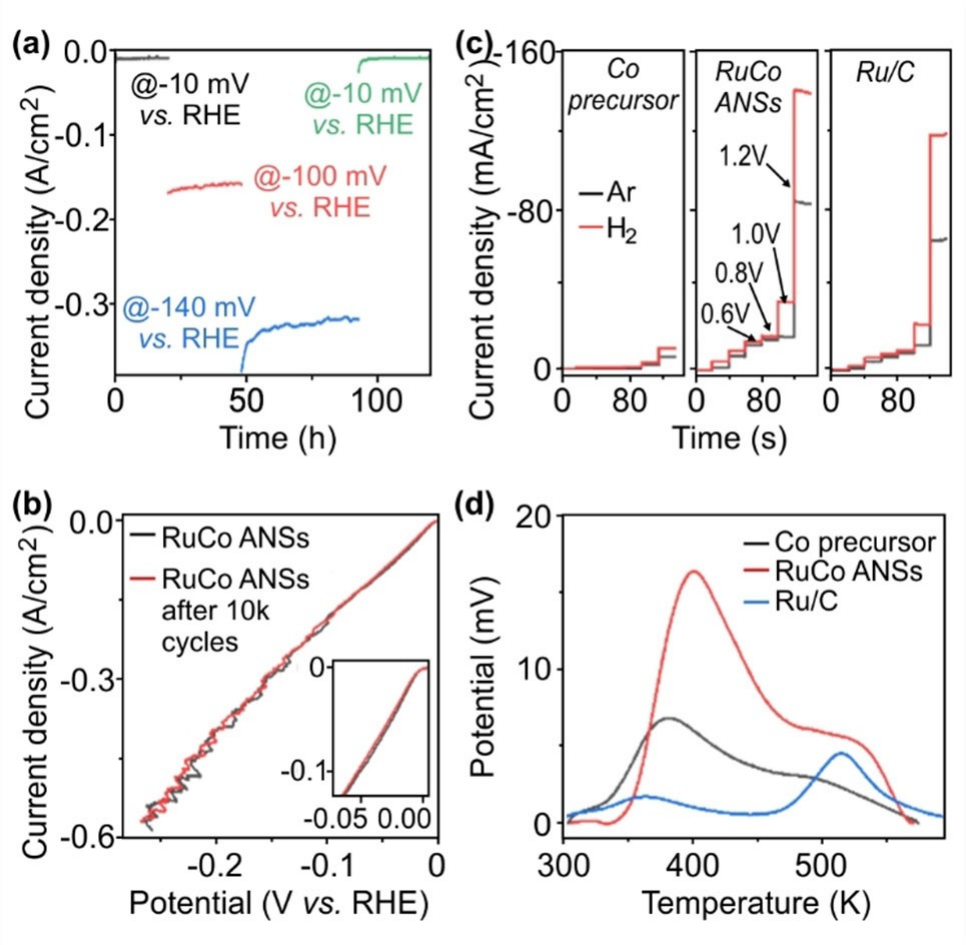
Catalytic stability and Ru−H binding characterization. Accelerating degradation tests of RuCo ANSs by chronoamperometry (a) and cyclic voltammetry (b). Inset in b shows a zoom‐in between −0.1 and 0 V vs. RHE. Hydrogen sensor (c) and TPD (d) tests of Co precursor, RuCo ANSs, and Ru/C.

To clarify the relationship between the structure stability and activity stability of RuCo ANSs, we analyzed the microstructure of RuCo ANSs after HER. The SEM image demonstrates the retained two‐dimensional layered structure of RuCo ANSs (Figure S15). Figure S16 shows the high‐resolution STEM image of the Co (100) facet, which is consistent with that in Figure 2e. Ru and Co are still homogeneously distributed in the RuCo ANSs (Figure S17). The XRD patterns show no obvious change before and after HER (Figure S18). The Co 2p and Ru 3p XPS spectra show that the chemical state of RuCo ANSs remains the same after HER (Figure S8). This is also verified by the Raman spectra in Figure S9. These results fully demonstrate the high structure stability of RuCo ANSs in alkaline HER.

To further investigate the relationships between Ru−H binding strength and HER activity, hydrogen sensor and temperature programmed desorption (TPD) tests were performed. As shown in Figure 4c, the sensor results reveal that the RuCo ANSs have the highest current difference of 17.7 mA cm^−2^ at −1 V, larger than the 1.5 mA cm^−2^ of the RuCo precursor, and the 10 mA cm^−2^ of commercial Ru/C. The TPD results (Figure 4d) show that RuCo ANSs possess a Ru−H bonding response up to 17 mV at ca. 400 K, larger than the one in the RuCo precursor (8 mV) and the commercial Ru/C (2 mV). These results demonstrate that the hydrogen adsorption/desorption efficiency is strong at the Ru sites in RuCo ANSs, which are in good agreement with our theoretical calculations. To further verify the prediction, RuNi ANSs were prepared using a similar method. As shown in Figure S19, the RuNi ANSs sample shows a HER activity better than commercial Ru/C, but worse than RuCo ANSs, which is consistent with the theoretical calculation results (Figure 1d).

In this work, we have demonstrated an efficient alkaline HER catalyst of RuCo ANSs by regulating the Ru−H interaction. DFT theoretical studies reveal that the interaction between the $4d_{z^{2}}$ orbital of Ru and the 1s orbital of H determines the Ru−H binding energy. The xy planar symmetric and Z‐direction asymmetric coordination structure of Ru sites on the Co nanosheets make it possible to accurately optimize the Ru $4d_{z^{2}}$ direction electronic structure. Experimentally, the RuCo ANSs were prepared via a fast co‐precipitation method followed by a mild electrochemical reduction. Structure characterization reveals that the Ru atoms are embedded into the Co substrate as isolated active sites with the xy planar symmetric and Z‐direction asymmetric coordination structure. Hydrogen sensor and temperature program desorption tests demonstrate that the Ru−H interactions in RuCo ANSs are enhanced compared to those in pure Ru nanoparticles. The RuCo ANSs show very high HER catalytic activity, with a low overpotential 10 mV at 10 mA cm^−2^. The TOF value of RuCo ANSs is 8.528 s^−1^ at 100 mV, much better than commercial Pt/C and Ru/C. The RuCo ANSs also show high stability during HER even under complex working conditions. We believe that these findings could be a forward step toward highly active catalysts for various hydrogen‐involving applications, such as ammonia borane degradations, water–gas shift and methane steam‐reforming reactions.

## Author contributions

Dr. C. Cai conducted the experiments and composed the manuscript. K. Liu conducted the DFT calculations. P. Li and Dr. Y. Zhu conducted the TEM experiments. Q. Wang conducted the XPS, Raman, and N_2_ adsorption/desorption experiments. All authors wrote, read and took part in the manuscript preparation, analysis and discussion.

## Conflict of interest

The authors declare no conflict of interest.

## Supporting information

As a service to our authors and readers, this journal provides supporting information supplied by the authors. Such materials are peer reviewed and may be re‐organized for online delivery, but are not copy‐edited or typeset. Technical support issues arising from supporting information (other than missing files) should be addressed to the authors.

Supporting Information
