# Supplementary material for: Optimizing Hydrogen Binding on Ru Sites with RuCo Alloy Nanosheets for Efficient Alkaline Hydrogen Evolution
Source: Angew Chem Int Ed Engl. 2021 Dec 7;61(4):e202113664. doi: 10.1002/anie.202113664 (PMC9300137; doi:10.1002/anie.202113664)
Supplement: Supplementary file 1 — Supporting Information [file ANIE-61-0-s001.pdf]

Angewandte Chemie
Eine Zeitschrift der Gesellschaft Deutscher Chemiker

Supporting Information

Optimizing Hydrogen Binding on Ru Sites with RuCo Alloy Nanosheets for Efficient Alkaline Hydrogen Evolution

C. Cai, K. Liu, Y. Zhu, P. Li, Q. Wang, B. Liu, S. Chen, H. Li, L. Zhu, H. Li, J. Fu, Y. Chen, E. Pensa, J. Hu, Y.-R. Lu, T.-S. Chan, E. Cortés, M. Liu**

<u>**Experimental section**</u>

Theoretical calculations

All the DFT calculations were performed using the Vienna ab Initio Simulation package (VASP).[1] Electron-ion interactions were described using standard PAW potentials.[2] For the electron-electron exchange and correlation functional was described through the generalized gradient approximation (GGA) of revised Perdew–Burke–Ernzerhof (RPBE).[3] A plane-wave cutoff energy of 450 eV was used in all the computations. The electronic structure calculations were employed with a Fermi-level smearing of 0.1 eV for the slab systems. A Monkhorst–Pack mesh with 3 × 3 × 1 K-points was used for the Brillouin zone integration. The convergence thresholds of the energy and forces were set to be 1×10^{-5} eV and 0.02 eV $Å^{-1}$, respectively. The chemical-bonding analysis of the electronic properties of Ru-H is processed using Local Orbital Basis Suite Toward Electronic-Structure Reconstruction (LOBSTER) program.[4,5]

To explore the Gibbs free energy of hydrogen adsorption (ΔGH^*) on Ru site, we calculated the ΔGH on the Ru slab hollow and top site. We found that the ΔG_{H^*} for the Ru hollow and top site are -0.22 and 0.13 eV, respectively, indicating weak adsorption of H* on Ru top site restrict HER. To optimize the adsorption of H* on Ru, we construct the model of Ru atom embedded in the Co and Ni slab. A (3×3×4) supercell containing 71 Co atoms and a Ru atom. Vacuum layer is set to 15 Å. The Gibbs free energy of each elementary step was calculated as

$$\Delta G = \Delta E + \Delta ZPE - T \bullet \Delta S$$

where ΔE is the reaction energy calculated by the DFT method. ΔZPE and ΔS are the changes in zero-point energies and entropy during the reaction, respectively.

Chemicals

All the chemicals are directly used in experiments without further purification. $Co(NO)_3\cdot6H_2O$ (>98%), cetyltrimethylammonium bromide (CTAB, >95%), $RuCl_3\cdot xH_2O$ (> 37%, Ru basis), sodium borohydride (SB, >99%), isopropanol (>95%), acetone (>95%), and KOH (>99.999%) were purchased from Aldrich. Nafion solution was purchased from sigma (5 wt.% aqueous solution). Commercial Pt/C, Ru/C is purchased from Macklin Biochemical (20 wt.%).

Synthesis of pure Co precursors

This experiment was carried out at room temperature in air. 0.6 mmol $Co(NO)_3\cdot6H_2O$ was added into 100 mL water (contains 15 mmol CTAB) with magnetic stirring to form solution A. After 20 min, 3 mmol SB in 20 mL water was added into solution A slowly. The system was held for another 1 h with magnetic stirring. The precipitation is washed by water and acetone for 5 times. Those black powders are dried in vacuum oven at 353 K for whole night. The precipitation is collected as Ru1Cox precursors.

Synthesis of RuCo precursors

This experiment was carried out at room temperature in air. 0.6 mmol $Co(NO)_3\cdot6H_2O$ and 1.2 mmol $RuCl_3\cdot xH_2O$ were added into 100 mL water (contains 15 mmol CTAB) with magnetic stirring to form solution A. After 20 min, 3 mmol SB in 20 mL water was added into solution A slowly. The system was held for another 1 h with magnetic stirring. The precipitation is washed by water and acetone for 5 times. Those black powders are dried in vacuum oven at 353 K for whole night. The precipitation is collected as Ru_1Co_x precursors.

Synthesis of RuCo ANSs

Rotate glassy disk carbon electrode (GCE) (3 mm) is the support of active materials to be used as working electrode. Ag/AgCl (saturated with 4 M KCl) and graphite rod were used as counter electrode and reference electrode, respectively. Electrolyte is 1 M KOH. Working electrode for Ru_1Co_x precursors was prepared by drop 5 uL dispersion (catalyst ink (2 mg/mL) with water containing 10 uL 5 wt. % Nafion solution) on a GCE (polished by Al_2O_3). The working electrode was applied in cyclic voltammetry (CV) from -1.0 to -1.824 V *vs*. RHE for 600 cycles with scan rate of 0.05 V/s. The working electrode was gently washed by water for several times and used for following characterizations.

Synthesis of RuNi ANSs

Similar to the synthesis process of Ru_1Co_x precursors without Co use, $Ni(NO_3)_2\cdot6H_2O$ is used as metal source for RuNi synthesis. Feeding ratio of Ru/Ni is 1:2.

Structural characterization

The morphology and microstructure characterization of the as-prepared samples were investigated by scanning electron microscopy (SEM) (FEI, Helios Nanolab 600i, 2kV), Transmission electron microscopy (TEM) and selected area electron diffraction (SAED) (FEI, ETEM, G2, 200kV), scanning transmission electron microscopy with an energy dispersive X-ray spectroscopy attachment (STEM-EDS, FEI Titan Themis, 300 kV). X-ray diffraction (XRD) (Bruker ECO D8 power X-ray diffractometer with Cu Kα radiation) were used to determine the crystal structure of the samples. The *in-situ* formed samples were dissolved into 10 mL nitric acid (1 M), and then the mixed solution was sonicated for 3 min. This solution was heated to 180 ºC for 2 h, and then the pink solution was collected for ICP-OES test. Hydrogen sensor test is conducted at room-temperature using a home-made instrument. In the Ar saturated container, the

current response curves at different potentials were collected. 5 % H_2/Ar was injected into container to get a H_2/Ar saturated container, in which, the H_2 adsorption/desorption response curves were collected. The TPD was conducted on PCA-1200.

Electrochemical characterization

All experiments were carried out using CHI760e and a three-electrode system in a PTFE bottle (100 mL). The *in-situ* formed working electrode (Ru_1Co_x ANSs) was tested at a spin rating of 1,600 rpm (Pine). Linear sweep voltammetry (LSV) and chronoamperometry were measured at 298 K, in air, and at 10 mV/s (scan rate) using the CHI 760e potentiostat. After tested at various density current, the catalyst was washed with water and ethanol to be measured by STEM, XPS, and Raman. The electrolyte was collected for ICP-OES testing. The cyclic voltammograms used for stability test were recorded using a scan rate of 0.05 V/s. The EIS was conducted on a Princeton 4000A at 100 mV overpotential from 100,000 to 0.01 Hz.

TOF calculations

The TOF value is calculated based on the ICP results, using the following equations:

$$TOF\ value = \frac{n_{H_2}\ (mol)}{n_{catalytic\ atom}\ (mol)\ \times t\ (s)}$$

where $n_{catalytic\ atom}$ is the number of catalytic atoms, t is time and n_{H_2} is the number of H_2 molecules, that are obtained from the electrochemical current (i) as:

$$n_{H_2}\ (mol) = \frac{i\ (A) \times t(s)}{F(\frac{C}{mol}) \times 2 \times \mathrm{n}_{catalysts\ loading}\ (mol)}$$

where i is the current, t is the time, F is the Faraday constant (96485 C/mol), 2 represents the number of electrons involves and $\mathrm{n}_{catalysts\ loading}$ is the amount of catalysts loading.

Figures and Tables

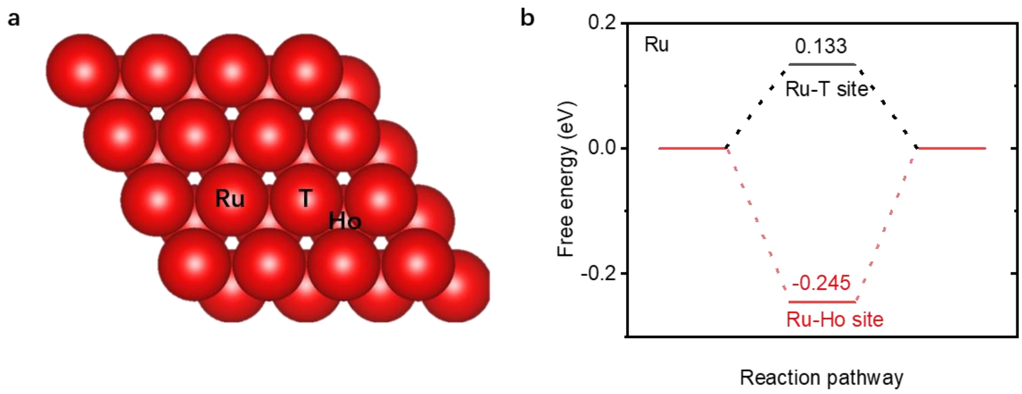

Figure S1. (a) Different hydrogen adsorption sites on Ru. T and Ho represent the Top and Hollow sites, respectively. (b) Free energy changes of hydrogen adsorption/desorption process at different sites of Ru. The Top site corresponding to vertical direction (Z direction) of Ru is responsible for the HER.

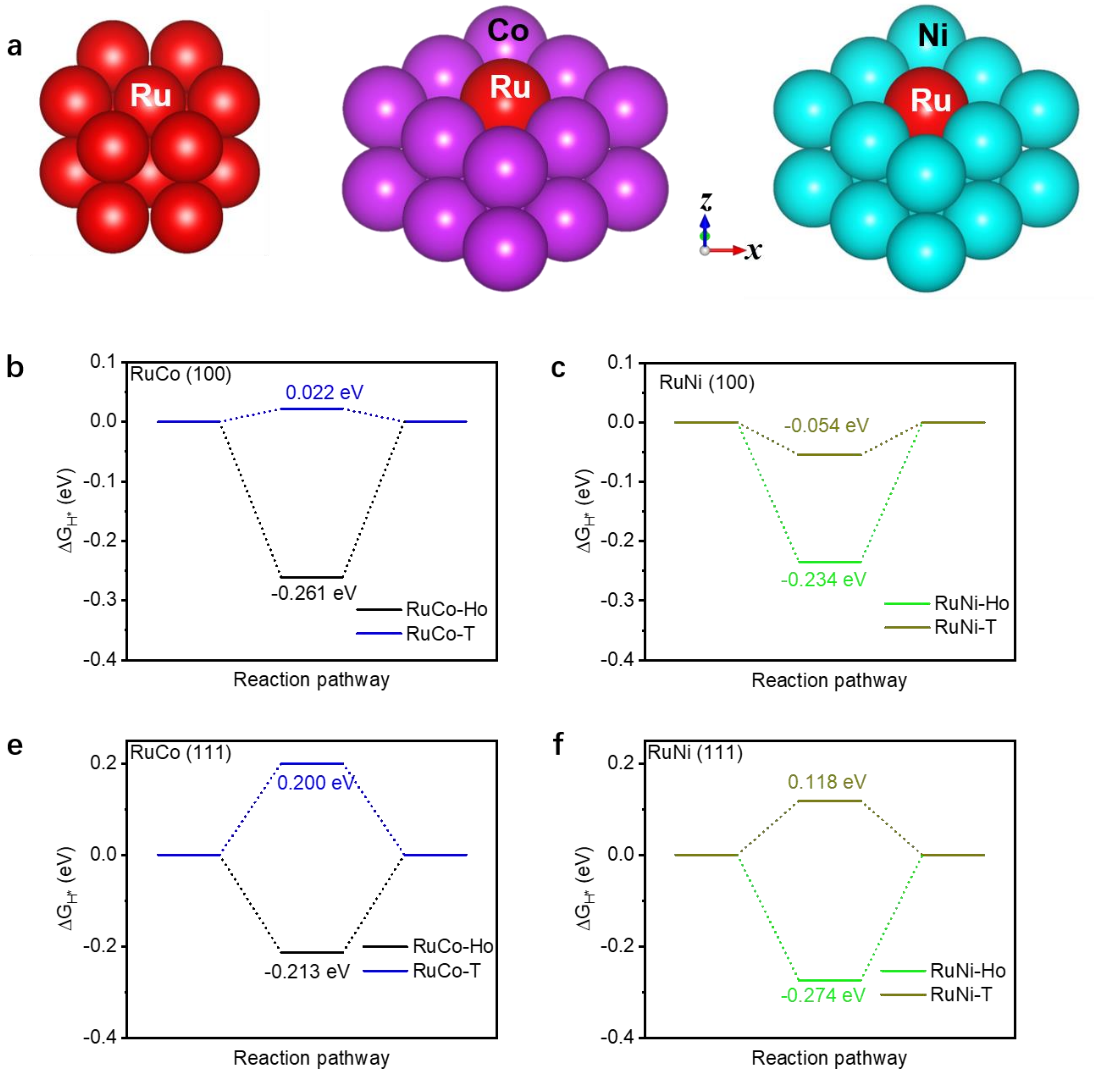

Figure S2. (a) Constructed models of Ru-M (M=Ru, Co, Ni) with symmetry in the xy plane and asymmetry in the Z direction. The comparison of ΔG_{H^*} for the different adsorption sites (T and Ho sites) of (b) RuCo(100), (c) RuNi(100) (c) RuCo(111) and (d) RuNi(111) .

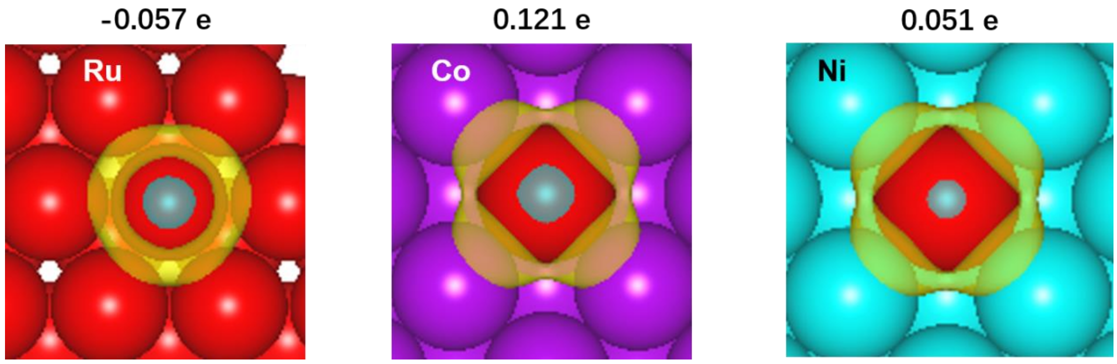

Figure S3. The charge transfer between the Ru site and substrate in the different models with exposed (100) facet. From left to right: Ru, RuCo and RuNi.

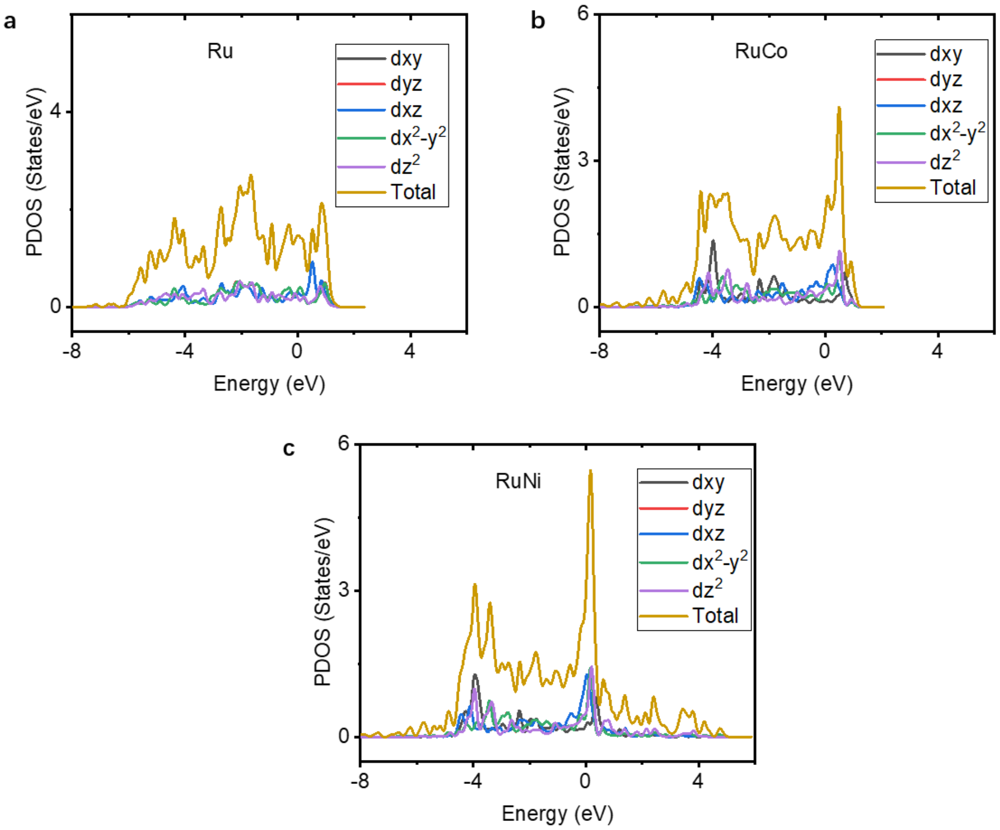

Figure S4. The PDOS of Ru 4d on (a) Ru(100), (b) RuCo(100) and (c) RuNi(100).

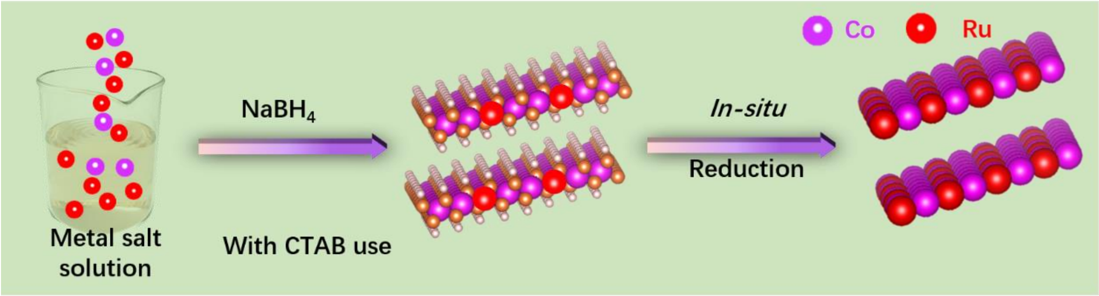

Figure S5. Scheme of the RuCo ANSs preparation process.

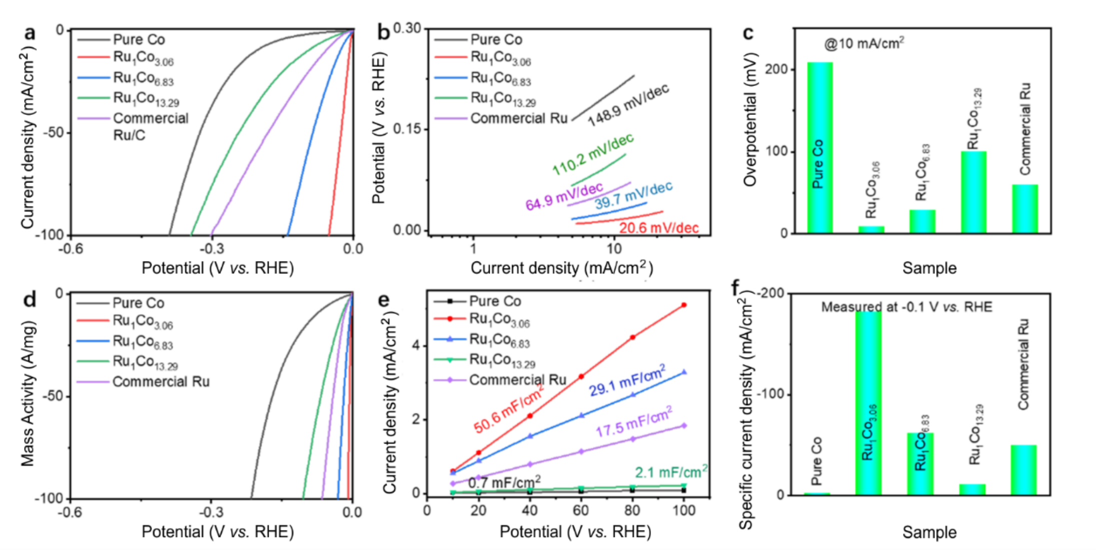

Figure S6. Electrochemical HER performance of Ru_1Co_x ANSs (Sample x=2, 4, 8 with compositions defined in Table S1) with different amount of Ru doping. (a) LSV curves, (b) Tafel plots, (c) specific overpotential, (d) mass activity, (e) C_{dl} value, and (f) mass activity at -1.1 V vs. RHE of Ru_1Co_x ANSs.

Table S1. The Ru/Co ratio of Ru_1Co_x ANSs defined by ICP-OES.

Feeding amount (Ru/Co)	Co (mg/L)	Ru (mg/L)	Molar ratio (Ru/Co)
**Ru_1Co_2 ANSs**	3.3777	1.8682	1:3.06
**Ru_1Co_4 ANSs**	6.9337	1.7217	1:6.83
**Ru_1Co_8 ANSs**	13.9374	1.7772	1:13.29
**Ru_1Co_2 ANSs after HER (electrolyte)**	0.0014	0.0011	1:2.27

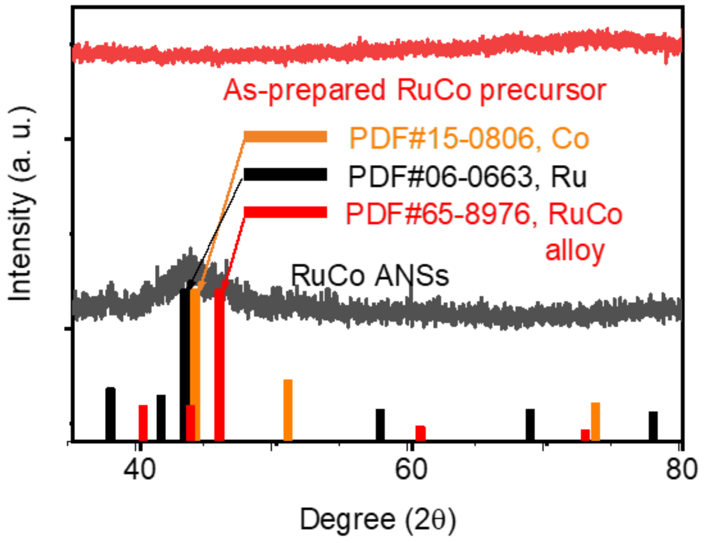

Figure S7. The XRD pattern of the as-prepared RuCo precursor (top, red) and RuCo ANSs (bottom, grey). Reference values are included in orange, black and red bars.

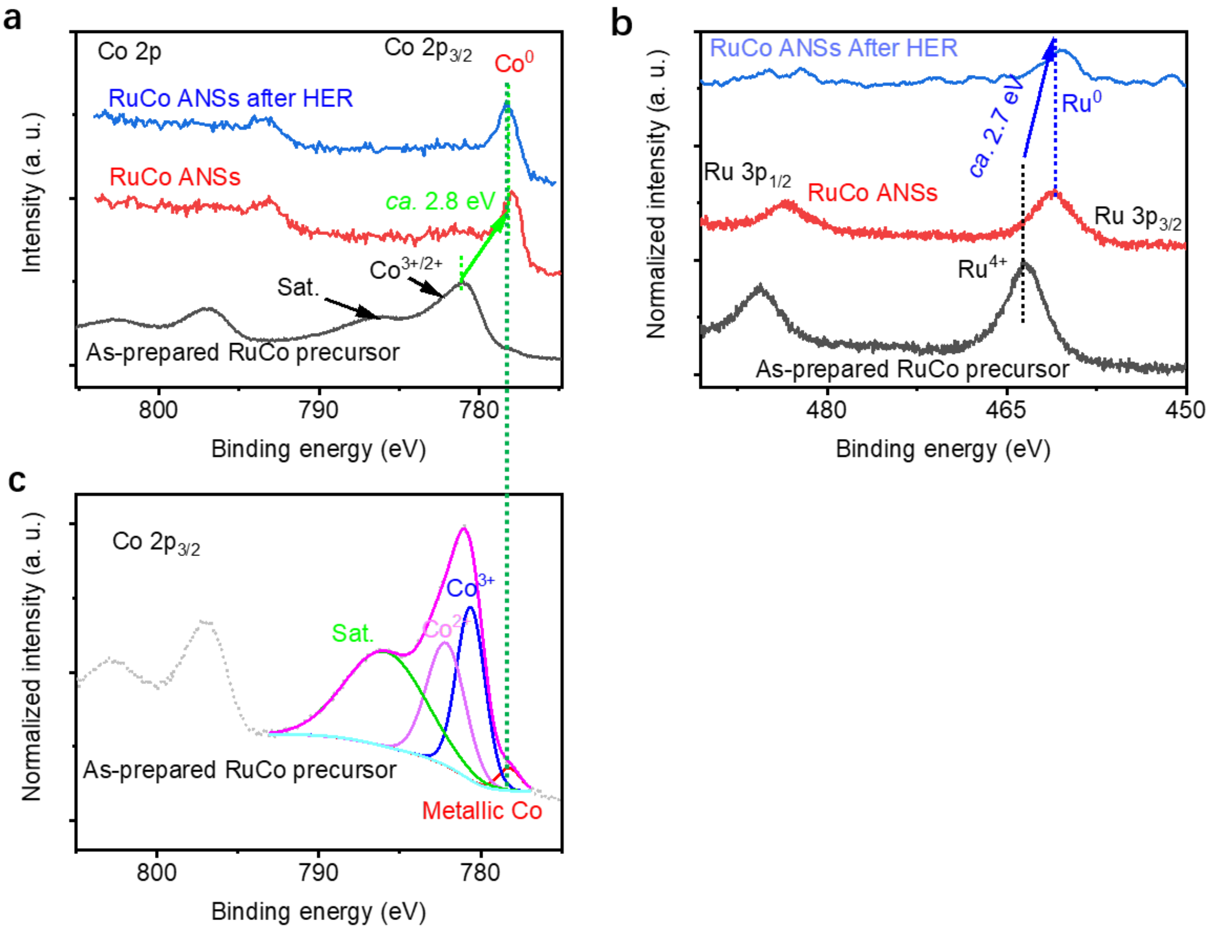

Figure S8. The (a) Co 2p and (b) Ru 3p XPS spectra of the as-prepared RuCo precursor (top, blue) and RuCo ANSs before (middle, red) and after (bottom, black) HER. (c) Deconvoluted Co2p spectrum. Metallic Co phase formation is observed after reduction of the RuCo precursor. The HER process does not affect the Co and Ru oxidation states.

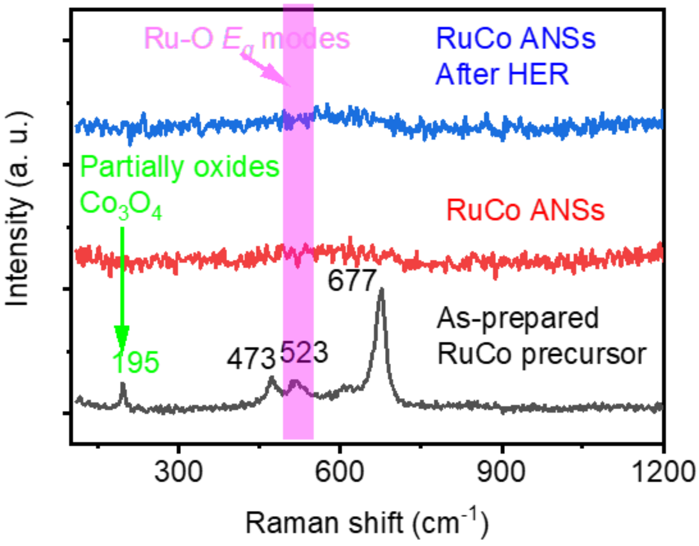

Figure S9. Raman spectra of the as-prepared RuCo precursor (bottom, black), RuCo ANSs (middle, red) and RuCo ANSs after HER (top, blue).The peaks located at 473 and 677 cm^{-1} belong to $Co(OH)_2$.[6] The small peak at 195 cm^{-1} belongs to Co_3O_4 phase, which may be caused by air oxidation. The peak located at 523 cm^{-1} is assigned to the E_g modes of Ru-O.[7] This peak vanishes after HER due to the metallic Ru formation.

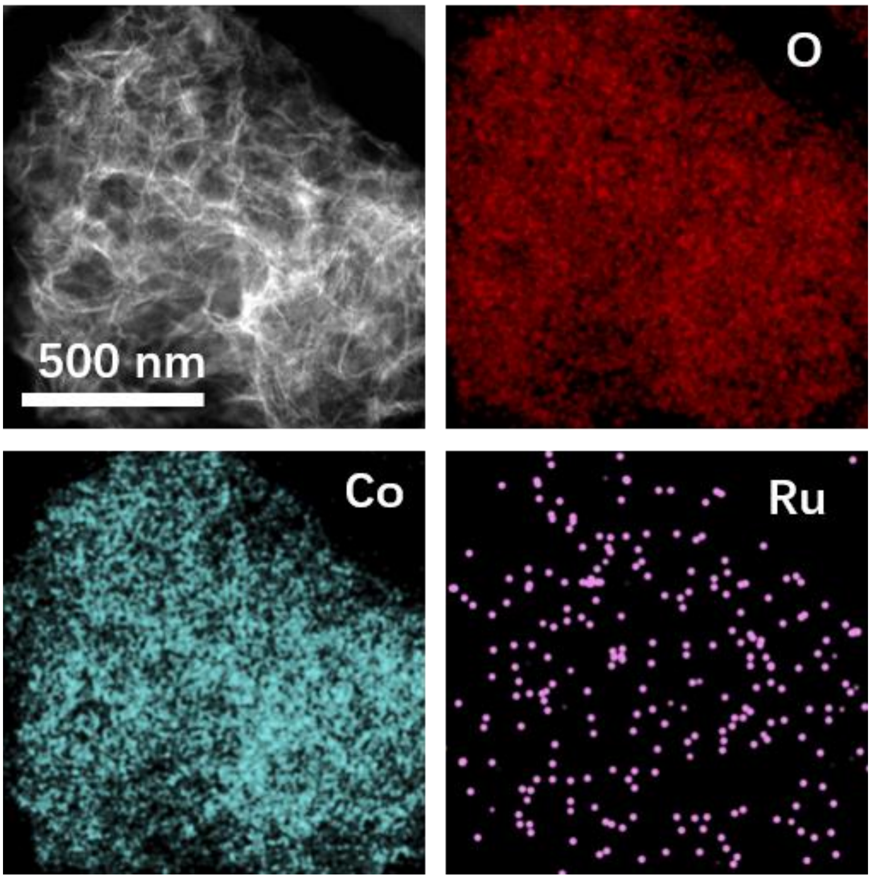

Figure S10. The STEM-EDS mapping of as-prepared RuCo precursor.

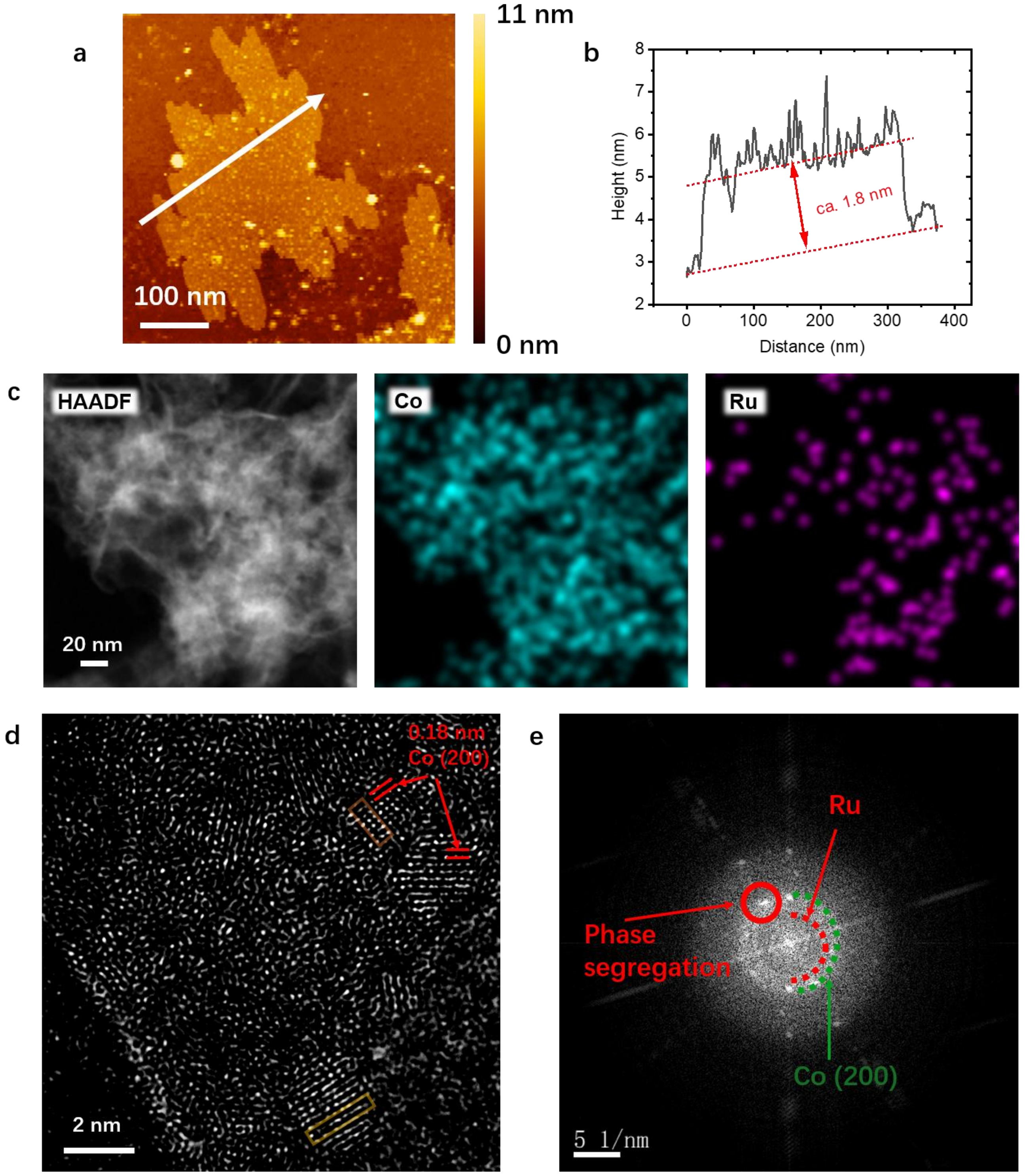

Figure S11. The AFM image (a), corresponding line profile (b), STEM-EDS mapping (c), HAADF image (d), and corresponding FFT results of the *in-situ* formed RuCo ANSs. The Phase segregation phase near to Co can be RuCo alloy signal.

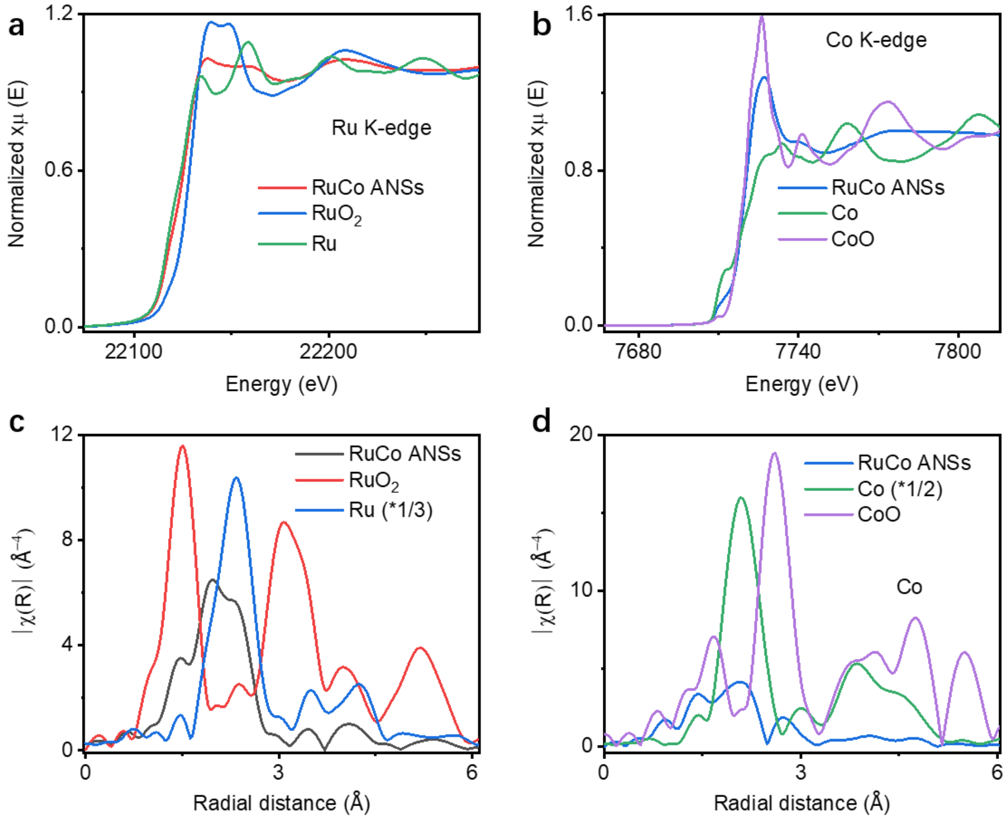

Figure S12. X-ray absorption near edge structure (XANES) of (a) Ru and (b) Co K-edge. FT-EXAFS of (c) Ru and (d) Co with their corresponding references. The red shift of white line in Ru (a) and Co (b) K-edge demonstrate the metallic phase formation of Ru and Co. This is consistent with the XRD and TEM results.

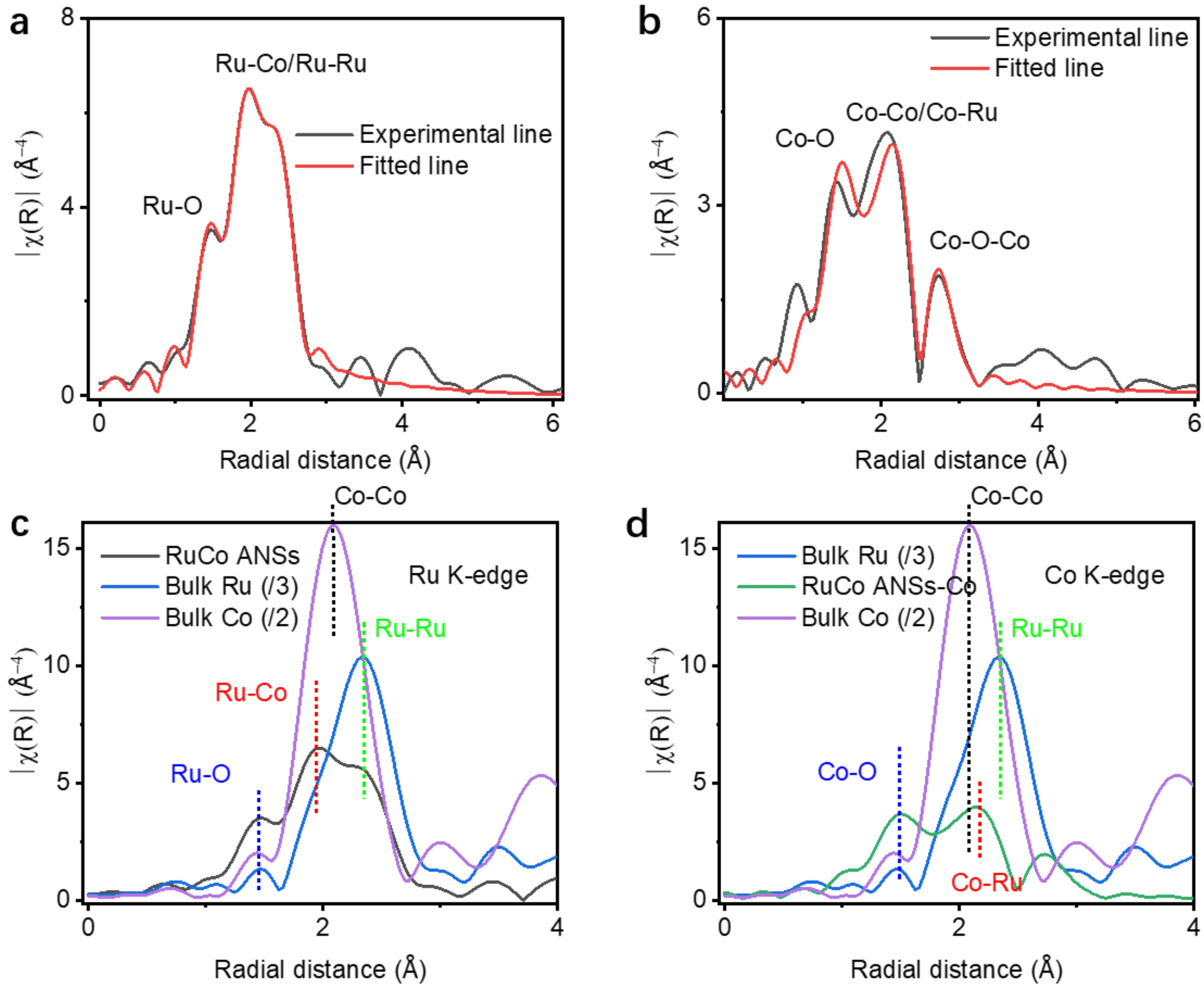

Figure S13. Fitted (a) Ru K-edge and (b) Co K-edge of RuCo ANSs. Peak comparison of Ru K-edge (c) and Co K-edge (d). Combing the RuCo ANSs and Bulk Co results in Figure S13c, we can see that no peak belongs to pure Co can be detected. By comparing the bulk Ru and RuCo ANSs in Figure S13d, we conclude that the peak located at radial distance of 2 Å belongs to Ru-Co peaks. Futhermore, in the Co K-edge, the Co-Ru peak can also be observed. In short, the EXAFS results demonstrate the RuCo alloy formation.

Table S2. Fitting parameters of the EXAFS spectra of RuCo ANSs. (CN: coordination number; R: distance; σ_2: mean-square disorder; E_0: energy shift).

Sample	Scattering Path	CN	σ^2(Å^2)	E_0 (eV)	R (Å)	R-factor
RuCo ANSs	Ru-O	1.878	0.00738	0.446	2.02531	0.0031803
	Ru-Co	2.544	0.00738	-8.967	2.54112	
	Ru-Ru	2.799	0.00738	-6.171	2.64492	
	Co-O	2.616	0.01015	-2.307	2.00399	0.0191579
	Co-O-Co	1.315	0.01024	-2.307	3.10389	
	Co-Co	1.734	0.01015	-2.307	2.52263	
	Co-Ru	0.163	0.00052	-2.307	2.64253	

Table S3. The performance comparison between Ru-based, Co-based, and Pt-based catalysts for alkaline HER (1 M KOH).

Materials	Overpotential (@ 10 mA/cm^2)	Tafel slope (mV/dec)	TOF of Ru @ overpotential	Mass activity (Ru) (A/g)	References
**Ru/Carbon-quantum-dots**	10 mV	47	/	/	[8]
**Ru/C hollow sphere**	18 mV	47	0.25 s^{-1}@15 mV	/	[9]
**CoRu0.25@N-C**	27 mV	53	/	/	[10]
**$CoRu_{0.5}$/Carbon-quantum-dots**	18 mV	38.5	/	/	[11]
**RuNi/ Carbon-quantum-dots**	13 mV	45	10 s^{-1}@200 mV	1.68 A/mg @13 mV	[12]
**Ru@SC-CDs**	29 mV	57	0.56 s^{-1}@100 mV,	/	[13]
**RuAu single atom alloy**	24 mV	37	2.18 s^{-1} @ 50 mV	/	[14]
**Ru@C_3N_4/C**	79 mV	Near to Pt	4.2 s^{-1}@100 mV	/	[15]
**Pt–$Co(OH)_2$/Ag**	29 mV	35.7	/	/	[16]
**Ru@Mo_2S**	13 mV	60	/	/	[17]
**Ru/CoO**	55 mV	70	/	/	[18]
**RuCo ANSs**	**10 mV**	**20.6**	**8.52 s^{-1}@100 mV**	**121 A/mg @13 mV**	**This work**

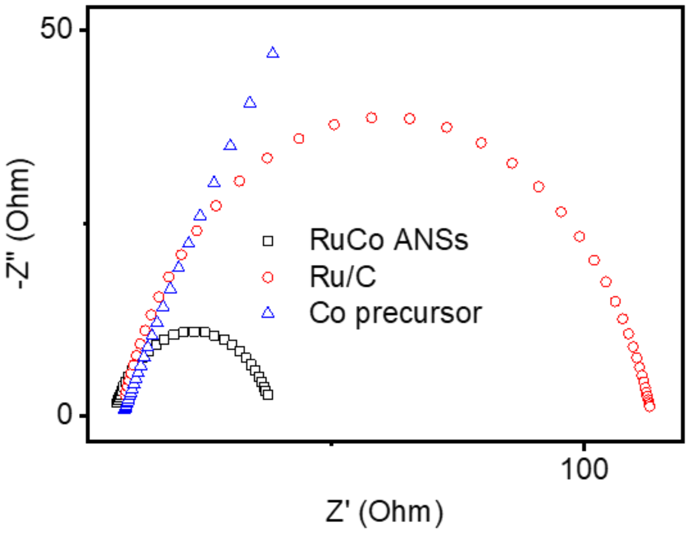

Figure S14. Nyquist representation of impedance spectra recorded for the as-prepared Co precursor (blue triangles), RuCo ANSs (black squares) and Ru/C (red circles).

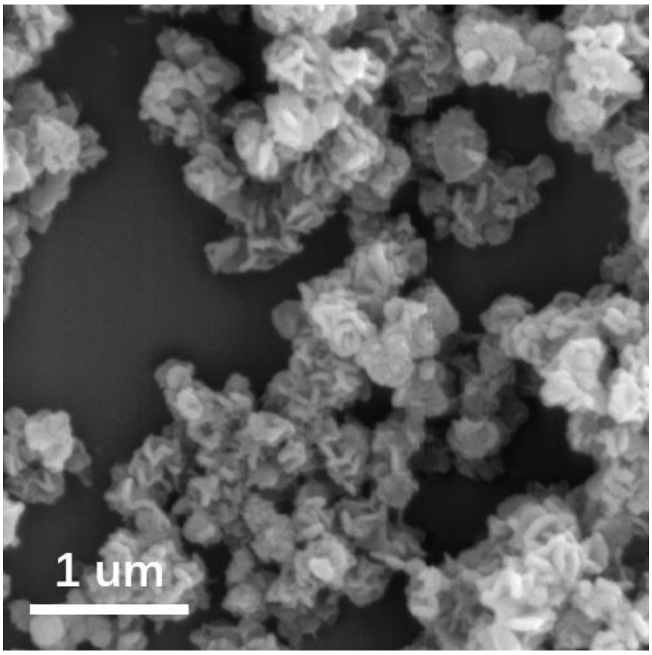

Figure S15. SEM image of RuCo ANSs after HER.

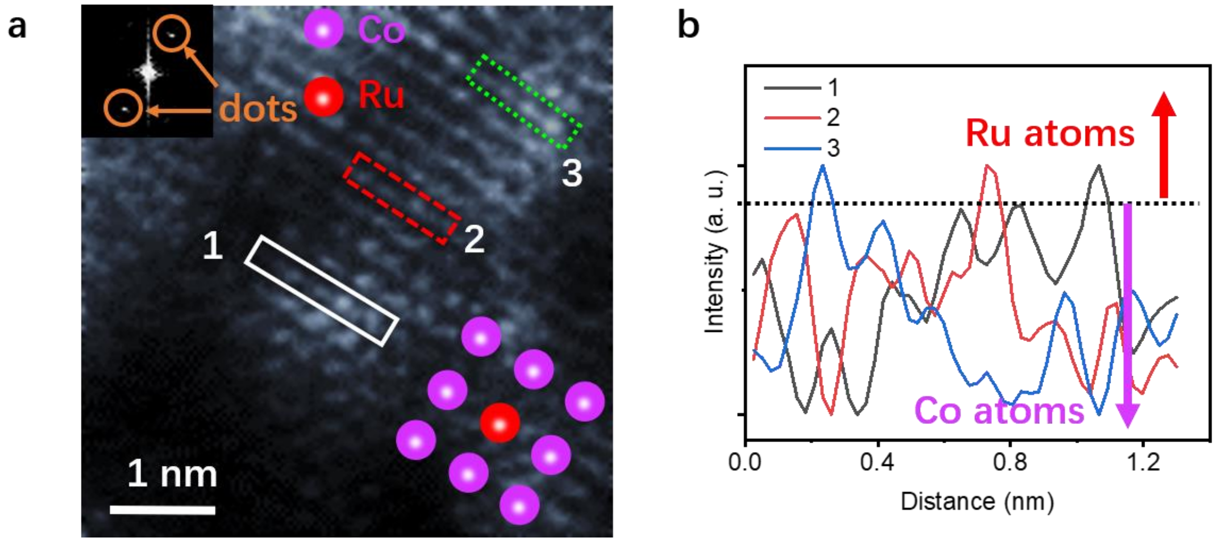

Figure S16. (a) Atomic resolution image and (b) relative profiles of RuCo ANSs after HER.

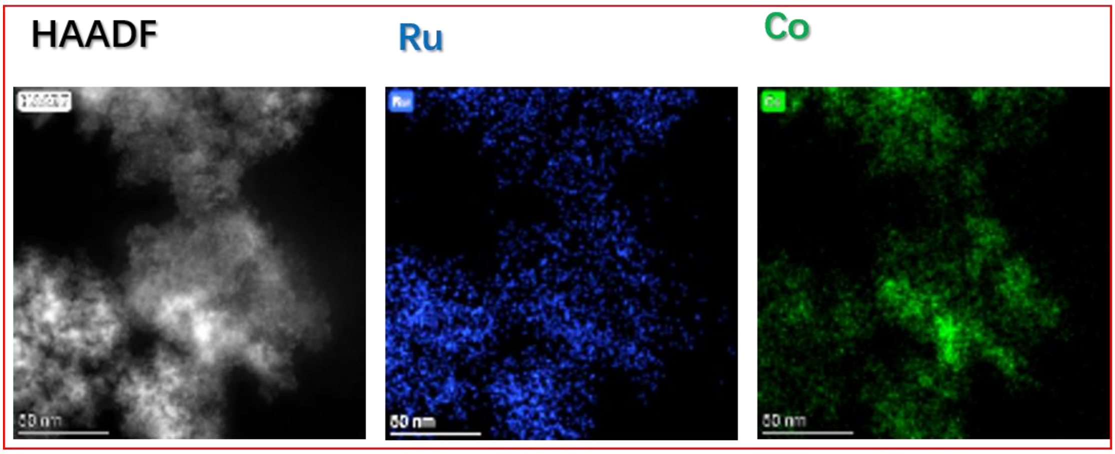

Figure S17. STEM-EDS of RuCo ANSs after HER.

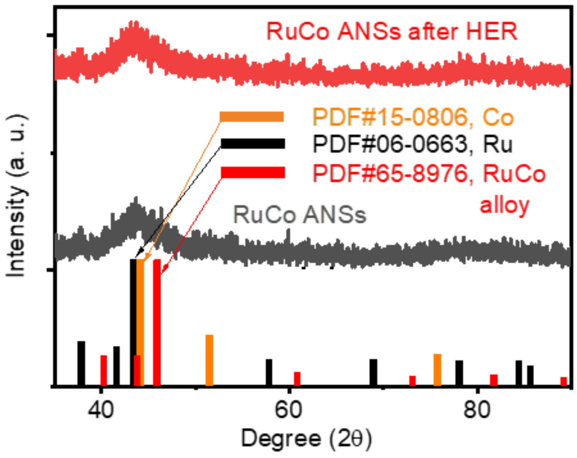

Figure S18. XRD patterns of RuCo ANSs before (bottom, black) and after (top, red) HER.

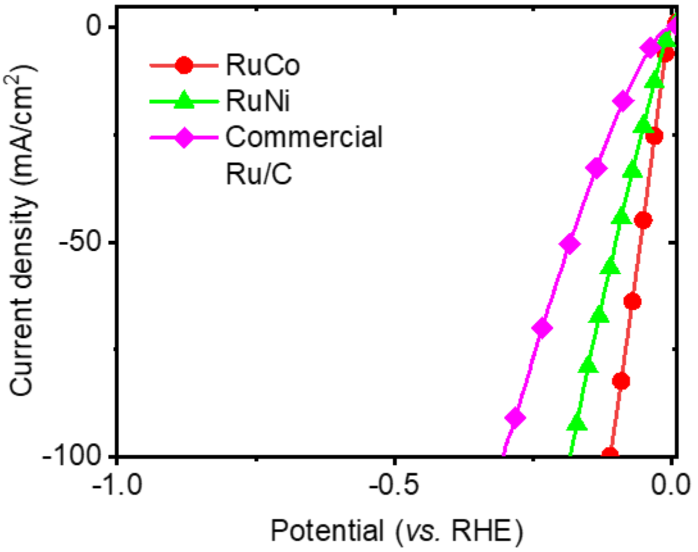

Figure S19. Comparison of HER performance for RuCo ANSs (red circles), RuNi ANSs (green triangles) and commercial Ru/C (pink diamonds).

References

[1] Kresse, Furthmuller, *Phys. Rev. B* **1996**, *54*, 11169-11186.
[2] Blochl, *Phys. Rev. B* **1994**, *50*, 17953-17979.
[3] B. Hammer, L. B. Hansen, J. K. Norskov, *Phys. Rev. B* **1999**, *59*, 7413-7421.
[4] V. L. Deringer, A. L. Tchougreeff, R. Dronskowski, *J. Phys. Chem. A* **2011**, *115*, 5461-5466.
[5] R. Nelson, C. Ertural, J. George, V. L. Deringer, G. Hautier, R. Dronskowski, *J. Comput. Chem.* **2020**, *41*, 1931-1940.
[6] M. A. Sayeed, T. Herd, A. P. O'Mullane, *J. Mater. Chem. A* **2016**, *4*, 991-999.
[7] S. Y. Mar, C. S. Chen, Y. S. Huang, K. K. Tiong, *Appl. Surf. Sci.* **1995**, *90*, 497-504.
[8] W. Li, Y. Liu, M. Wu, X. Feng, S. A. T. Redfern, Y. Shang, X. Yong, T. Feng, K. Wu, Z. Liu, B. Li, Z. Chen, J. S. Tse, S. Lu, B. Yang, *Adv. Mater.* **2018**, *30*.
[9] Z. Peng, H. Wang, L. Zhou, Y. Wang, J. Gao, G. Liu, S. A. T. Redfern, X. Feng, S. Lu, B. Li, Z. Liu, *J. Mater. Chem. A* **2019**, *7*, 6676-6685.
[10] Z. Wei, Y. Liu, Z. Peng, H. Song, Z. Liu, B. Liu, B. Li, B. Yang, S. Lu, *ACS Sustain. Chem. Eng.* **2019**, *7*, 7014-7023.
[11] W. Li, Y. Zhao, Y. Liu, M. Sun, G. I. N. Waterhouse, B. Huang, K. Zhang, T. Zhang, S. Lu, *Angew. Chem. Int. Ed. Engl.* **2020**.
[12] Y. Liu, X. Li, Q. Zhang, W. Li, Y. Xie, H. Liu, L. Shang, Z. Liu, Z. Chen, L. Gu, Z. Tang, T. Zhang, S. Lu, *Angew. Chem. Int. Ed. Engl.* **2020**, *59*, 1718-1726.
[13] Y. Liu, Y. Yang, Z. Peng, Z. Liu, Z. Chen, L. Shang, S. Lu, T. Zhang, *Nano Energy* **2019**, *65*.
[14] C.-H. Chen, D. Wu, Z. Li, R. Zhang, C.-G. Kuai, X.-R. Zhao, C.-K. Dong, S.-Z. Qiao, H. Liu, X.-W. Du, *Adv. Energy Mater.* **2019**, *9*.
[15] Y. Zheng, Y. Jiao, Y. Zhu, L. H. Li, Y. Han, Y. Chen, M. Jaroniec, S.-Z. Qiao, *J. Am. Chem. Soc.* **2016**, *138*, 16174-16181.
[16] K. L. Zhou, C. Wang, Z. Wang, C. B. Han, Q. Zhang, X. Ke, J. Liu, H. Wang, *Energy Environ. Sci.* **2020**, *13*, 3082-3092.
[17] J. Liu, Y. Zheng, D. Zhu, A. Vasileff, T. Ling, S. Z. Qiao, *Nanoscale* **2017**, *9*, 16616-16621.
[18] J.-X. Guo, D.-Y. Yan, K.-W. Qiu, C. Mu, D. Jiao, J. Mao, H. Wang, T. Ling, *J. Energy Chem.* **2019**, *37*, 143-147.
